# Marburg virus disease outbreak in Guinea: a SPIN framework of its transmission and control measures for an exemplary response pattern in West Africa

**DOI:** 10.11604/pamj.2021.40.143.31709

**Published:** 2021-11-08

**Authors:** Frankline Sevidzem Wirsiy, Denis Ebot Ako-Arrey, Claude Ngwayu Nkfusai, Eugene Vernyuy Yeika, Luchuo Engelbert Bain

**Affiliations:** 1 Pfizer Scholar_One Young World (OYW), London, United Kingdom,; 2 Cameroon Society of Epidemiology (CaSE), Yaoundé, Cameroon,; 3 IntraHealth Uganda, Kampala, Uganda,; 4 Department of Public Health, School of Nursing and Public Health, University of Kwa-Zulu Natal, Durban, South Africa,; 5 Impact Santé Afrique, Yaoundé, Cameroon,; 6 Global South Health Research and Services (GSHS), Amsterdam, The Netherlands,; 7 Ministry of Public Health, Yaoundé, Cameroon,; 8 Lincoln International Institute for Rural Health (LIIRH), College of Social Science, University of Lincoln, Lincoln, Lincolnshire, United Kingdom

**Keywords:** Marburg virus, outbreak, Guinea, transmission, SPIN framework

## Abstract

Responding to highly infectious diseases relies on a thorough understanding of transmission epidemiology. With the recent outbreak of Marburg Virus Disease (MVD) in Guinea, we saw the need to shed some technical light based on published literature and our field experiences. We reviewed 14 previous MVD outbreaks globally. Coupled with core one - health approaches, we propose a Socio-environmental context, Possible transmission routes, Informing and guiding public health action, Needs in terms of control measures (SPIN) framework as a guiding tool for response teams to appropriately approach future infectious disease outbreaks.

## Commentary

Responding to highly infectious diseases which involve their prevention and control; relies on a thorough understanding of the epidemiological factors enhancing transmission [1]. Also, minimizing the transmission of infectious diseases remains a core function of the public health principles. MVD is a highly virulent disease; caused by the Marburg virus (MARV) that was first recognized in 1967 [2] and a recent outbreak has been established in Guinea. The reservoir host of the Marburg virus has been established to be the African fruit bat - *Rousettus aegyptiacus* [3]. However, fruit bats infected with the Marburg virus do not show obvious signs of illness [2]. Humans can become infected with the Marburg virus, and may develop serious diseases with high mortality. MVD fatality rates reported in different outbreaks ranged from 24-100% [4], even though the disease generally occurs at a low prevalence. Clinically, MVD causes a severe hemorrhagic fever, known as Marburg hemorrhagic fever (MHF), in both nonhuman and humans primates [5]. It is worthy to note that, Marburg virus, is a genetically animal-borne/unique zoonotic ribonucleic acid (RNA) virus of the filovirus family (Ebola virus are the only other known members of the filovirus family). Two large outbreaks that occurred simultaneously in Marburg and Frankfurt in Germany, and Belgrade, Serbia, in 1967, led to the initial recognition of the disease [6]. The outbreak was associated with laboratory work using African green monkeys imported from Uganda; a country prone to MVD outbreaks. After the discovery, there have been a total of 14 MVD outbreaks as seen in Table 1, most of which occurred in Africa, with a few outbreaks occurring outside Africa even though traced back to Africa [6]. The history of the outbreaks guided the conception of the SPIN framework (Table 1).

**Table 1:** fourteen Marburg virus disease outbreaks (1967 to 2021)

No	Year and country	Suspected and/or apparent origin	The apparent situation of MVD infection (sociodemographic characteristics, circumstances warranting suspicion, diagnosis, and source of MVD infection)
1	2021, Guinea	Guéckédou, Guinea	A male, had onset of symptoms in a small health facility near his village of residence with symptoms of fever, headache, fatigue, abdominal pain, and gingival hemorrhage; the patient received supportive care and eventually died in the community; the team collected a post-mortem oral swab sample, for conducting real-time PCR which confirmed the sample was positive for MVD
2	2017, Uganda	Kween, Uganda	A blood sample from a patient in Kween District in Eastern Uganda tested positive for MARV; within 24 hours of confirmation, a rapid outbreak response was begun; the index case was a herdsman who hunted games around caves harboring enormous populations of the Egyptian fruit bats
3	2014, Uganda	Kampala, Uganda	Overall, one case was confirmed (fatal) and 197 contacts were followed for 3 weeks but all tested negative at the Uganda Virus Research Institute (UVRI)
4	2012, Uganda	Kabale, Uganda	Testing at CDC/UVRI identified a Marburg virus disease outbreak in the districts of Kabale, Ibanda, Mbarara, and Kampala over 3 weeks
5	2008, Netherlands ex Uganda	Cave in Maramagambo forest in Uganda	A Dutch woman with a recent history of travel to Uganda was admitted to a hospital in the Netherlands; three days before hospitalization, the first symptoms (fever, chills) occurred, followed by rapid clinical deterioration after which the woman died of MVD
6	2008, USA ex Uganda	Cave in Maramagambo forest in Uganda	A United States traveler that returned from Uganda was retrospectively diagnosed with MVD
7	2007, Uganda	Lead and gold mine in Kamwenge District, Uganda	Four young males working in a mine in Uganda were tested positive for MVD
8	2004-2005, Angola	Uige Province, Angola	This MVD's largest outbreak is believed to have begun in Uige Province of Angola in October 2004; so far, in the history of MVD outbreaks, this outbreak recorded the highest number of infected cases (252) of which 227 (90%) died; the majority of cases detected in other provinces in Angola have been linked directly to the outbreak in Uige; there was a lag in outbreak identification and poor epidemiological linkage of the cases, resulting in uncertainties as to identifying the origin of the infection
9	1998-2000, Democratic Republic of Congo (DRC)	Durba, DRC	The MVD cases occurred in young male workers at a gold mine in Durba, in the north-eastern part of the Democratic Republic of Congo
10	1990, Russia	Russia	The MVD case was a result of laboratory contamination; in this laboratory, there was a non-compliance with safety conditions and/or manipulation error when handling the virus resulting in causing human contamination in Russia in 1990; it is worthy to note that, an infected person remains contagious after his death, thus touching such required appropriate personal protective equipment
11	1987, Kenya	Kenya	An MVD infected Danish boy who later died had visited the Kitum Cave in Mount Elgon National Park in Kenya; no further cases were detected
12	1980, Kenya	Kenya	The case was that of an MVD male patient that died and had a history of recent travel to Kenya; while in Kenya, he visited the Kitum Cave in Mount Elgon National Park; a doctor who attempted resuscitation developed symptoms 9 days later but recovered
13	1975, Johannesburg, South Africa	Zimbabwe	The primary case was in a young Australian man who eventually died with a recent travel history to Zimbabwe for tourism with his spouse; the circumstances regarding their infection suggest that there was likely a direct contact with bats discharge as they slept in rooms containing insectivorous bats which led to the infection; the infection spread from the man to his traveling companion and a nurse at the hospital (nosocomial infection), even though they recovered
14	1967, Germany and Yugoslavia	Uganda	Simultaneous outbreaks occurred in laboratory workers handling African green monkeys imported from Uganda; in addition to the 31 reported cases, an additional primary case was retrospectively serologically diagnosed and other cases were reported due to nosocomial transmission; also, a woman got infected with MVD, 3 months earlier via sexual contact with her husband

Taking a summary look at Table 1, the transmission dynamics of MARV is such that, source of infection includes errors in laboratory experiments for example during the MVD outbreak in Germany and Yugoslavia simultaneously, when laboratory workers experimenting on grivets (African savanna green monkey) imported from Uganda were infected with MARV after handling tissues/organs of these monkeys. Also, other cases were reported due to nosocomial transmission, and retrospectively, a woman who got infected with MVD, 3 months earlier via sexual contact with her husband. Other major outbreaks occurred as a result of tourism (for example holiday safari in Uganda, where activities included visiting local and viewing wildlife in villages and camping), working in mines, hunting around caves harboring enormous populations of the Egyptian fruit bats.

Marburg haemorhagic fever has been typically appearing in sporadic outbreaks throughout Africa with laboratory-confirmed cases have been reported in Guinea, Uganda, South Africa, Zimbabwe, the Democratic Republic of the Congo, Angola, and Kenya. However, isolated and/or sporadic MVD cases may occur, but go unrecognized. This necessitates an efficient surveillance system. Moreover, with the recent outbreak of Marburg virus disease (MVD) in Guinea [7], amid the COVID-19 pandemic as well as the most recent Ebola virus disease (EVD) outbreak in Ivory Coast and that of Guinea that was declared over on June 19^th^, 2021, by the World Health Organization (WHO), there is need to highlight the risk of MARV transmission in the community as well as control measures that will serve to inform the response efforts against this outbreak.

**Figure 1:**
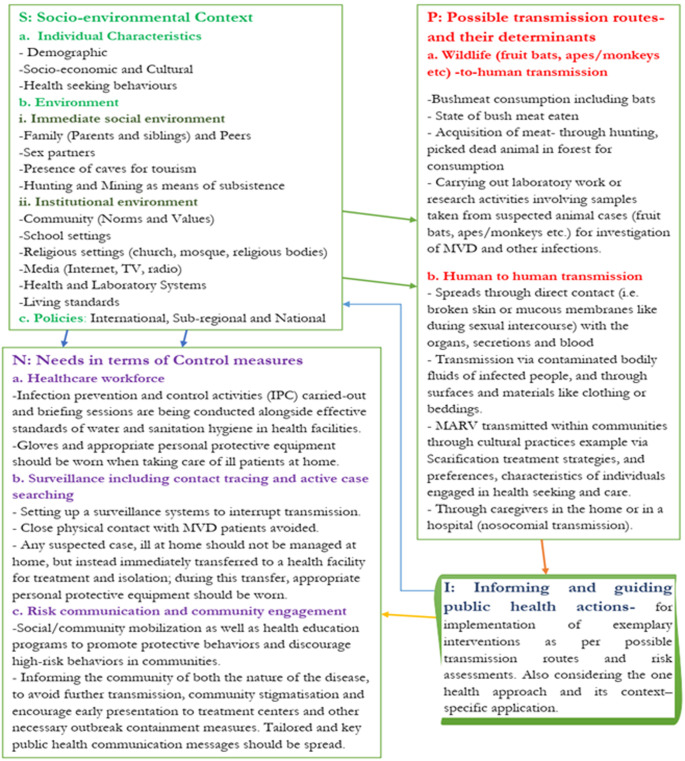
a socio-environmental context, possible transmission routes, informing and guiding public health action, needs in terms of control measures (SPIN) framework of transmission and control measures of an MVD outbreak

We developed the SPIN (Socio-environmental context, Possible transmission routes, Informing and guiding public health action, Needs in terms of control measures) framework (Figure 1) in consideration to the history of 14 MVD outbreaks that have been recorded which including the context involving the individual characteristics of target communities, the environment in which they find themselves and the policies in place that directly/indirectly affects their needs (control measures). The SPIN framework is composed of four key elements i.e. the socio-environmental context (made up of individual characteristics, environment, policies); possible transmission routes, and their determinants (made up of wildlife (fruit bats, apes/monkeys, etc) -to-human transmission and human to human transmission); informing and guiding public health actions for implementation of exemplary interventions and control measures (Figure 1).

**Epidemiological transmission routes of Marburg virus disease:** with the perspective of the one health concept, there is an increasing tendency of zoonoses [8], since animal habitats are frequently visited by humans partly because of tourism and/or exploration, animal habitat destruction, and hunting for bushmeat consumption, thus outcome being increased contact between wildlife and humans. Wildlife (fruit bats, apes/monkeys, etc) -to-human transmission, has been evident to be a source of transmission for the Marburg virus [9]. In the same light, handling wildlife in conjunction with hand hygiene and personal protective equipment such as gloves and other appropriate protective clothing is crucial, without which transmission can easily occur [6]. Animal products that have not been cooked thoroughly before consumption is a possible transmission route of MARV and as such should be avoided, talk less of consumption of raw meat [2].

Tourism, that is visits in caves or mines that are inhabited by fruit bat colonies, need to be monitored as they have been established as means through which MARV is transmitted [1]. Infection with MVD is associated with prolonged exposure to caves/mines inhabited by Rousettus bats [10]. When carrying out laboratory work or research activities, scientists are also at risk of transmission as samples were taken from suspected cases (humans) and animals (fruit bats, apes/monkeys) for investigation of Marburg infection can transmit this virus [10].

After this initial crossover of virus from host animal to humans, transmission occurs through human-to-human contact such that, the person infected with MARV, spreads through direct contact (i.e. broken skin or mucous membranes) with the organs, secretions, blood, or other contaminated bodily fluids of infected people, and surfaces and materials like clothing or beddings [2]. Furthermore, Filoviruses´ MARV and Ebola can be transmitted within communities through cultural practices (via scarifications), under-protected family care settings, and under-protected health care staff [11], as there is direct contact to infectious droplets of body fluids of patients or indirect contact with objects contaminated with infectious tissues/blood. Another instance is through caregivers in the home or a hospital (nosocomial transmission).

**Control of Marburg virus disease:** MVD infection can be avoided by not been in contact with sick non-human primates and fruit bats, in West Africa, can protect. Key strategies to control an MVD outbreak include setting up a surveillance system to interrupt transmission and social/community mobilization as well as health education programs to promote protective behaviors and discourage high-risk behaviors in communities [2]. Active surveillance has been shown to enhance the rapid identification and isolation of symptomatic patients, to reduce the likelihood of transmission to others, and then treatment [10]. Case investigations, as part of surveillance activities, identifying probable sources of infection and contacts of the suspected cases is necessary. This data obtained in turn, can inform health education messages and interventions around high-risk behaviors in the community and facilitate monitoring of contacts during the MVD incubation period (2-21 days), i.e. contact tracing, allowing for their rapid isolation and treatment should symptoms develop [10].

An exemplary MVD outbreak control should equally involve a range of interventions such as setting up event-based surveillance, a good laboratory-systems service, safe and dignified burials. The improvement in the use of laboratory diagnostic tools is essential for MVD control. Also, it is very important to reduce the risk of human-to-human transmission from direct or close contact with people with Marburg symptoms, particularly with their bodily fluids [10]. Gloves and appropriate personal protective equipment should be worn when taking care of ill patients at the hospital [5]. Washing the hands regularly is of utmost importance after physical contact and visiting patients in health care facilities, or those being taken care of at home. In the same light, reducing the risk of possible sexual transmission, involve male or female condom use. Washing the body with water and medicated soap and is essential in the fight against MVD.

## Conclusion

In rapidly responding to MVD outbreaks, we can use the SPIN framework to enhance our understanding of the potential and primary sources of MVD infection to avoid a lag in outbreak identification including poor epidemiological linkage of the probable cases which can result in uncertainties to identifying the origin of the infection and thus increasing spread of the outbreak. In essence, taking into consideration the SPIN framework, it will inform targeted interventions exemplary response patterns in Guinea, West Africa, and the whole African continent at large. Even though MHF is and remains a rare human disease, it's very dangerous. However, when it occurs, it has the potential to rapidly spread to other people, especially with health care staff and family members who care for the suspected, probable, or confirmed MVD patient. Therefore, having the SPIN framework in mind together with reinforcing awareness in communities as well as among healthcare providers of the clinical symptoms of patients with MHF is critical. Better awareness can lead to earlier and stronger precautions against the spread of MARV in both family members, healthcare providers, and our communities in general.
